# Effect of opium consumption on cardiovascular diseases – a cross- sectional study based on data of Rafsanjan cohort study

**DOI:** 10.1186/s12872-020-01788-4

**Published:** 2021-01-02

**Authors:** Parvin Khalili, Fatemeh Ayoobi, Maryam Mohamadi, Ahmad Jamalizadeh, Carlo La Vecchia, Ali Esmaeili-nadimi

**Affiliations:** 1 Department of Epidemiology, School of Public Health, Iran University of Medical Sciences, Tehran, Iran; 2 Non-Communicable Diseases Research Center, Rafsanjan University of Medical Sciences, Rafsanjan, Iran; 3 Pistachio Safety Research Center, Rafsanjan University of Medical Sciences, Rafsanjan, Iran; 4 Health System Research Center, Rafsanjan University of Medical Sciences, Rafsanjan, Iran; 5 Department of Clinical Sciences and Community Health, University degli Study di Milano, Milan, Italy

**Keywords:** Ischemic heart diseases, Myocardial infarction, Opium use, Prospective epidemiological research studies in IrAN (PERSIAN)

## Abstract

**Background:**

There are differences of opinion about the beneficial or detrimental effects of opium consumption on cardiovascular diseases (CVDs). So, we aimed to study the association between opium use and CVDs.

**Methods:**

We used data obtained from the Rafsanjan Cohort Study (RCS), as a part of the prospective epidemiological research studies in IrAN (PERSIAN), with detailed, validated data on opium consumption and some other exposures. A total of 10,000 adults were enrolled in the study. Logistic regression models were used to assess the possible relationships of opium consumption with the prevalence of ischemic heart diseases (IHD) and myocardial infarction (MI).

**Results:**

In this study, 9990 participants in the baseline phase of the Rafsanjan adult cohort study were included according to their completed questionnaire. Among all participants, 870 and 296 individuals were found to suffer from IHD and MI, respectively. Opium consumption was found to be relatively high in the RCS participants, especially in men (men = 2150 and women = 228). Opium use was associated with a higher odds of IHD and MI, with the adjusted odds ratios (95% CI) of 1.51 (1.22–1.86) and 1.79 (1.31–2.45), respectively. Also, dose-response increases were observed with the highest odds ratios in the 4th quartile for MI and IHD (*p*-values for trend < 0.001). Increased odds were observed for the two main methods of opium consumption, i.e. oral and smoking, but oral administration had higher odds ratio.

**Conclusions:**

Opium consumption is associated with the increased odds of both IHD and MI diseases.

## Background

Opium addiction is common in many countries globally. This problem is more common in Middle Eastern countries, especially Pakistan, Afghanistan, and Iran [1, 2]. In 2018, the United Nations Office of Drugs and Crime (UNODC) estimated that 53 million people of the world population (5.5%) aged between 15 and 64, had consumed drugs in the previous year [3, 4] and 585,000 people died as a result of drug use in 2017 [4]. Opium, along with its derivatives, is the most common drug abused in Iran [5]. In recent years, few cases on lead poisoning due to opium consumption in Iran have been reported [6]. Additionally, some researchers reported the contamination of opium with lead in the southeast of Iran [7, 8]. This toxic substance exists as an impurity and adulterant in the illicit opium distributed in Iran. Lead has severe effects on human health [9].

Prevalence of cardiovascular diseases (CVDs) showed an increase in recent years [10]. It has been reported that CVDs are the cause of 50% of deaths in developed countries and 25% of deaths in developing countries [11]. According to the report of World Health Organization (WHO), CVDs cause one third of all annual deaths worldwide [12]. For a long time, it was believed that opium use can prevent the traditional risk factors of CVDs and equilibrate metabolic systems [13]. Particularly, some of healthcare incumbents and even physicians conceived that opium consumption has a preventive role on insulin resistance, diabetes mellitus, and lipid profile disturbances [14]. For instance, according to a large clinical survey in 2015, around 5.2% of the Iranian patients that suffered from CVDs were opium-dependent [15]. It was a result of a misconception that opium consumption might reduce the adverse impacts of CVDs [15]. The effects of opium use on the cardiovascular system have been mediated by some endogenous ligands such as opioid peptides [16]. Although, some people still assume that opium can be an alternative medication for cardiovascular risk factors, especially diabetes [13], several studies showed that opium addiction increased the levels of homocysteine that in turn results in the increased risk of CVDs in addicted population [17, 18]. Also, some other studies indicated that opium addiction is a risk factor for CVDs [19, 20].

The purpose of the present study was to investigate the relation of the duration and route of opium consumption with ischemic heart diseases (IHD) and myocardial infarction (MI) CVDs.

## Methods

### Study design and patient selection

This cross-sectional study was conducted on participants of Rafsanjan cohort study (RCS); as a part of the prospective epidemiological research studies in IrAN (PERSIAN) [21], launched in August 2015 in Rafsanjan, a region in south east of Iran. Study population was selected via 9990 sampling that had complete habit history [22]. Study inclusion criterion was the age range of 35–70 years (male or female). Study protocol was designed according to the Persian cohort study and was approved by the Ethics Committee of Rafsanjan University of Medical Sciences (Ethical codes: ID: IR.RUMS.REC.1399.081).

### Data collection

All participants underwent a standardized interview to completely validated questionnaires containing questions on demography, socioeconomic status, smoking behavior, opium use, alcohol consumption, history of disease, blood pressure, body mass index (BMI) and physical activity. Questionnaires were validated in the PERSIAN cohort study [21]. In this study, opium use was defined as self-reported use of opium. Subjects were divided into two groups of opium non-users (ONUs = 7612 subjects), opium users (OUs = 2378 subjects) [22].

CVD prevalence was assessed using self-reported information from the medical history questionnaire. Prevalent CVDs was defined as IHD and MI based on the self-reporting of the participants that a doctor told them they had angina, a myocardial infarction or reported undergoing coronary bypass surgery, balloon angioplasty or stent placement in coronary arteries [22].

Fasting serum total cholesterol, high density lipoprotein cholesterol (HDL cholesterol), low density lipoprotein cholesterol (LDL cholesterol), S.G.O.T (AST), S.G.P.T (ALT), alkaline phosphatase, and triglycerides were measured using a CPALS analyzer (Coultronics, Margency, France) at the Central Laboratory in Cohort center.

### Exposure and other covariate assessment

To assess opium use, we used a structured questionnaire in which detailed questions about age at the time of starting opium use, amount and frequency of use (e. g. how many days a week in the case of weekly use), administration routes, opium types, and age the time of quitting for those who had quitted opium use. Routes of administration included opium smoking and oral consumption. Opium types included teriak, Sukhteh, and Shireh. Teriak is a sticky paste which is prepared after air-drying the raw opium [23, 24]. Sukhteh is a black dry residue which sticks to the opium pipe after smoking Teriak. Sukhteh is then scraped from the pipe and can be ingested. Shireh is a refined product of opium which is often obtained by boiling a combination of raw opium and Sukhteh in water and filtering the mixture several times. Heroin is another product obtained from opium [23, 24]. However, its use among the participants of this cohort was rare, so heroin use was not evaluated in this study.

### Statistical analyses

The chi-square test was used to analyze categorical variables across opium use categories. t -test was used to compare continuous variables among the groups. Logistic regression models were used to investigate the relationships between opium use and the prevalence of IHD and MI. Confounders were identified using a causal diagram (Additional file 1: Figure 1) [25, 26]. Based on subject matter knowledge and the relevant epidemiological literature, these confounders were sequentially entered into models according to their hypothesized strengths of association with opium use and IHD and MI. To reach this goal, separate models at bivariate level were run to obtain variables associated with IHD and MI. Afterwards, variables with a *p*-value < 0.2 were considered for multivariate analysis [26]. Adjusted model 1 included basic sociodemographic characteristics (age, gender and education years) considered to be the most strongly related to both opium use and IHD and MI. Adjusted model 2 adjusted for lifestyle confounding variables (tobacco smoking, alcohol drinking) and physical activity level in addition to the sociodemographic characteristics, to additionally confound opium use – IHD and MI associations. Adjusted model 3 included all variables in adjusted model 2 and additionally included hypertension, hypercholesterolemia, body mass index and diabetes mellitus, triglycerides, LDL cholesterol, HDL cholesterol, S.G.O.T (AST), S.G.P.T (ALT), and alkaline phosphatase. As these were hypothesized to be potential intermediates on the causal pathways that could explain opium use – IHD and MI relationships. In all models, variables of age, education years, hypercholesterolemia, body mass index (BMI) and, triglycerides, LDL cholesterol, HDL cholesterol, S.G.O.T (AST), S.G.P.T (ALT), alkaline phosphatase were entered continuously. Also, for current users, duration of use was categorized into quintiles to test for dose-response relationships. Also, the data were analyzed by routes of administration of opium used. Notably, for assess the association between opium consumption and the CVDs, we excluded individuals who reported opium use after their illness. Finally, we excluded 38 patients (31 men and 7 female) who had started opium consumption after their illness.

Since opium use is categorized as stigmatized and sensitive behaviors, in this study some degree of non-differential misclassification is probable as a result of misreporting or recall bias. So, we performed a simple bias analysis, one of the quantitative bias analysis methods, and compared the result of this analysis with that of the conventional result, to determine the direction and magnitude of the misreporting bias [27].

To determine the bias parameters (sensitivity and specificity of self-reported opium use), the results obtained from an internal validity study can be used. However, when the resources to perform an internal validation study are not available, previously-published validation studies which are applicable to the obtained data should be used. Finally, in the case of the existence of no relevant published data, the researchers have to use their experience and estimate the classification parameters.

Since no internal validation study has been performed in this population to assess the self-reporting of opium use, we used the results of an external validation study [28] and guess based on the existing conditions [22] to determine the bias parameters and perform simple bias analysis in order to correct the exposure measurement error (refer to applying quantitative bias analysis to epidemiologic data book for more details on how a simple bias analysis is performed) [27].

All analyses were conducted in State V.12. All *p*-values are two-sided, and *p*-values < 0.05 and 95% confidence intervals were considered as statistically significant.

We run a simple bias analysis, one of the quantitative bias analysis methods,

## Results

In this study, 9990 participants in the baseline phase of the Rafsanjan adult cohort study were included. From this population, 4655 (46.60%) were male and 5335 (53.40%) were female. Overall, the biological samples of 9941 participants were collected on which laboratory measurements were carried out.

Table 1 shows the data collected from the opium users and non-users from the aspects of socio-demographic characteristics, lifestyle, personal habits, anthropometric measures, clinical risk factors and blood laboratory assessment. A participant is defined as opium user if he/she reports using opium for at least once per week for 6 months, prior to admission. The rate of opium use is relatively high in the RCS participants, especially in men (46.19% of men and 4.27% of women).

**Table 1:** Selected characteristics of the Rafsanjan cohort participants (*n* = 9990)

Status of opium consumption				
Characteristics	All (n = 9990)	Non-user (***n*** = 7612)	User (***n*** = 2378)	***P***-Value
Age - yr. Mean ± SD	49.91 ± 9.56	49.33 ± 9.57	51.78 ± 9.31	< 0.001
Age- no. (%)				< 0.001
35–44	3466 (34.69)	2833 (37.22)	633 (26.62)	
45–54	3041 (30.43)	2290 (30.08)	750 (31.54)	
55–64	2759 (27.62)	1984 (26.06)	775 (32.59)	
≥ 65	725 (7.26)	505 (6.63)	220 (9.25)	
Gender- no. (%)				< 0.001
Female	5335 (53.40)	5107 (67.09)	228 (9.59)	
Male	4655 (46.60)	2505 (32.91)	2150 (90.41)	
Education-no. (%)				< 0.001
Illiterate	948 (9.50)	707 (9.30)	241 (10.13)	
Elementary	2547 (25.52)	1961 (25.79)	586 (24.64)	
Guidance school	2479 (24.84)	1688 (22.20)	791 (33.26)	
Diploma	2368 (23.73)	1845 (24.27)	523 (21.99)	
College	1639 (16.42)	1402 (18.44)	237 (9.97)	
Physical activity Mean ± SD	38.77 ± 6.36	38.47 ± 5.59	8.29 ± 39.72	< 0.001
Alcohol consumption- no. (%)				< 0.001
yes	1351 (13.63)	469 (6.21)	882 (37.33)	
no	8561 (86.37)	7080 (93.79)	1481 (62.67)	
Cigarette smoking-no. (%)				< 0.001
Yes	2541 (25.66)	816 (10.82)	1725 (73.03)	
No	7362 (74.34)	6725 (89.18)	637 (26.97)	
Hookah smoking- no. (%)				< 0.001
yes	1706 (17.22)	969 (12.84)	737 (31.20)	
No	8202 (82.78)	6577 (87.16)	1625 (68.80)	
Hypertension- no. (%)				< 0.001
yes	2235 (22.50)	1823 (24.13)	412 (17.33)	
No	7698 (77.50)	5732 (75.87)	1966 (82.67)	
Cholesterol- no. (%)				< 0.001
> 200(mg/dL)	4279 (43.04)	3404 (44.91)	875 (37.06)	
≤ 200(mg/dL)	5662 (56.96)	4176 (55.09)	1486 (62.94)	
Mean ± SD	198.78 ± 41.89	200.66 ± 42.74	192.73 ± 38.43	< 0.001
Triglycerides- no. (%)				< 0.001
> 200(mg/dL)	2437 (24.53)	1779 (23.49)	658 (27.89)	
≤ 200(mg/dL)	7496 (75.47)	5795 (76.51)	1701 (72.11)	
Mean ± SD	168.88 ± 109.22	165.86 ± 107. 28	178.57 ± 114.71	< 0.001
LDL cholesterol - no. (%)				< 0.001
> 130(mg/dL)	2143 (21.56)	1718 (22.66)	425 (18.00)	
≤ 130(mg/dL)	7798 (78.44)	5862 (77.34)	1936 (82.00)	
Mean ± SD	108.04 ± 30.49	109.23 ± 30.36	104.21 ± 30.61	< 0.001
HDL cholesterol - no. (%)				0.029
< 35(mg/dL)	42 (0.42)	26 (0.34)	16(0.68)	
≥ 35(mg/dL)	9899 (99.58)	7554 (99.66)	2345 (99.32)	
Mean ± SD	57.90 ± 12.45	59.04 ± 12.67	54.24 ± 10.95	< 0.001
S.G.O.T (AST) - no. (%)				0.329
≤ 39(U/L)	286 (2.88)	225 (2.97)	61 (2.58)	
> 39(U/L)	9655 (97.12)	7355 (97.03)	2300 (97.42)	
Mean ± SD	19.87 ± 11.81	19.72 ± 11.62	20.36 ± 12.38	0.022
S.G.P.T (ALT) - no. (%)				0.052
≤ 39(U/L)	9130 (91.84)	6939 (91.54)	2191 (92.80)	
> 39(U/L)	811 (8.16)	641 (8.46)	170 (7.20)	
Mean ± SD	21.55 ± 15.37	21.74 ± 15.38	20.95 ± 15.32	0.030
Alkaline phosphatase- no. (%)				< 0.001
> 306 (IU/L)	972 (9.78)	663 (8.75)	309 (13.09)	
≤ 306 (IU/L)	8969 (90.22)	6917 (91.25)	2052 (86.91)	
Mean ± SD	225.48 ± 66.81	222.05 ± 65.86	236.50 ± 68.66	< 0.001
BMI- no. (%)				< 0.001
< 25	2894 (28.97)	1813 (23.82)	1081 (45.46)	
25–29.9	4089 (40.94)	3213 (42.22)	876 (36.84)	
≥ 30	3006 (30.09)	2585 (33.96)	421 (17.70)	
Mean ± SD	27.79 ± 4.99	28.40 ± 4.91	25.84 ± 4.76	< 0.001
Diabetes mellitus- no. (%)				0.005
yes	1933 (19.46)	1517 (20.08)	416 (17.49)	
no	8000 (80.54)	6038 (79.92)	1962 (82.51)	

*BMI* body mass index

There are differences in some habits, measured clinical and laboratory indices among opium user and non-user participants (Tables 1 and 2). Educational attainment is lower in the opium users. Physical activity, HDL, alcohol consumption and cigarette smoking are considerably higher in opium users. Hookah smoking is more common among the opium non-users. Abnormal serum levels of triglycerides, cholesterol, AST, and alkaline phosphatase are realized to be directly associated with opium using, whereas inverse association is observed between opium using and the abnormal serum levels of cholesterol, LDL cholesterol, and ALT. Furthermore, the frequency of hypertension, diabetes mellitus and obesity is significantly lower in the opium users.

**Table 2:** Prevalence of myocardial infarction and ischemic heart diseases among the participants in the Rafsanjan cohort study (n = 9990)

Characteristic	Myocardial infarction	***P*** -value	Ischemic heart diseases	***P*** -value
Opium consumption- no. (%)		< 0.001		< 0.001
yes	154 (52.03)		313 (35.98)	
no	142 (47.97)		557 (64.02)	
Duration of opium consumption - no. (%)				
Non-user	142 (47.97)	< 0.001	557 (64.02)	< 0.001
≤ 5 year	21 (7.1)		61 (7.01)	
6–10 year	21 (7.1)		52 (5.98)	
11/15 year	26 (8.78)		41 (4.71)	
16/20 year	35 (11.82)		56 (6.44)	
> 20 year	51 (17.23)		103 (11.84)	
Route of opium consumption - no. (%)		< 0.001		< 0.001
Smoking	140 (47.30)		282 (32.42)	
Oral consumption	14 (4.73)		31 (3.56)	
Age cat- no. (%)		< 0.001	0 (0.00)	< 0.001
35–44	18 (6.08)		56 (6.44)	
45–54	64 (21.62)		201 (23.10)	
55–64	157 (53.04)		433 (49.77)	
≥ 65	57 (19.26)		180 (20.69)	
Gender- no. (%)		< 0.001		0.003
Female	85 (28.72)		424 (48.74)	
Male	211 (71.28)		446 (51.26)	
Education-no. (%)		< 0.001		< 0.001
Illiterate	54 (18.24)		175 (20.11)	
Elementary	84 (28.38)		263 (30.23)	
Guidance school	54 (18.24)		180 (20.69)	
Diploma	62 (20.95)		158 (18.16)	
College	42 (14.19)		94 (10.80)	
Physical activity. Mean ± SD	36.66 ± 5.28	< 0.001	37.18 ± 5.28	< 0.001
Alcohol consumption- no. (%)		0.012		0.645
yes	55 (18.58)		114 (13.12)	
no	241 (81.42)		755 (86.88)	
Cigarette smoking -no. (%)		< 0.001		< 0.001
yes	152 (51.35)		286 (32.91)	
no	144 (48.65)		583 (67.09)	
Hookah consumption- no. (%)		0.060		0.065
yes	63 (21.28)		130 (14.96)	
no	233 (78.72)		739 (85.04)	
Hypertension- no. (%)		< 0.001		< 0.001
yes	119 (40.20)		440 (50.57)	
no	177 (59.80)		430 (49.43)	
Cholesterol- no. (%)		< 0.001		< 0.001
> 200(mg/dL)	86 (29.25)		269 (31.10)	
≤ 200 (mg/dL)	208 (70.75)		596 (68.90)	
Triglycerides- no. (%)		0.224		0.824
> 200 (mg/dL)	81(27.55)		215 (24.86)	
≤ 200(mg/dL)	213 (72.45)		650 (75.14)	
LDL cholesterol - no. (%)		0.103		0.043
> 130 (mg/dL)	52 (17.69)		163 (18.84)	
≤ 130 (mg/dL)	242 (82.31)		702 (81.16)	
HDL cholesterol - no. (%)		0.001		0.069
< 35 (mg/dL)	5 (1.70)		7 (0.81)	
≥ 35 (mg/dL)	289 (98.30)		858 (99.19)	
S.G.O.T (AST) - no. (%)		0.046		0.617
≤ 39 (U/L)	280 (95.24)		838 (96.88)	
> 39 (U/L)	14 (4.76)		27 (3.12)	
S.G.P.T (ALT) - no. (%)		0.530		0.034
≤ 39 (U/L)	273 (92.86)		811 (93.76)	
> 39 (U/L)	21 (7.14)		54 (6.24)	
Alkaline phosphatase- no. (%)		0.004		0.007
> 306 (IU/L)	43 (14.63)		107 (12.37)	
≤ 306 (IU/L)	251 (85.37)		758 (87.63)	
BMI- no. (%)		0.065		0.137
< 25	101 (34.12)		229 (26.32)	
25–29.9	121 (40.88)		359 ( ( (41.26)	
≥ 30	74 (25.00)		282 (32.41)	
Diabetes mellitus- no. (%)		< 0.001		< 0.001
yes	124 (41.89)		343 (39.43)	
no	172 (58.11)		527 (60.57)	

*BMI* body mass index

Among all participants, 8.76% IHD and 2.98% MI were reported (Table 2). Prevalence of IHD and MI is considerably more common among men (more than 2 times), especially in the age range of 55–64 years old. Both of the CVDs are more prevalent among opium users, cigarette smokers, overweighed people (BMI = 25–29.99) and sedentary people. Additionally, hypercholesterolemia and hypertension have direct association with the CVDs. As expected, consumption route and duration have significant effects on the prevalence of MI and IHD. Longer consumption duration of opium has resulted in the higher incidence of both CVDs. In the case of consumption route, MI and IHD are remarkably more common among opium smokers compared with those consume opium orally.

Table 3 presents the association of opium use with MI and IHD diseases, using the crude and three adjusted models. In the crude regression model, the odds of IHD (odds ratio (OR): 1.68, 95%CI 1.44 to 1.96) and MI (odds ratio (OR): 3.35, 95%CI 2.64 to 4.25) are almost Twice and triple greater among opium users compared with non-users. This association persisted after adjustment for confounders (adjusted model 2). The corresponding adjusted ORs calculated for opium users in comparison to non-users are 1.57 (95% CI 1.28 to 1.93) and 1.93 (95% CI 1.42 to 2.63), respectively for IHD and MI diseases. Adjusted model 3 includes all variables considered in adjusted model 2, plus hypertension, hypercholesterolemia, BMI, diabetes mellitus, triglycerides, LDL, HDL, AST, ALT, and alkaline phosphatase which could act as potential intermediates in the causal pathways describing the relationship of opium use with IHD and MI diseases. However, after adjusting for the variables (adjusted model 3), the obtained results showed no appreciable change and the mentioned association of IHD (odds ratio: 1.51, 95%CI 1.22 to 1.86) and MI (odds ratio (OR): 1.79, 95%CI 1.31 to 2.45) with opium use was observed again. When the results were divided by quartile of consumption duration in the current users, dose-response increases were observed with the highest odds ratios in the 4th quartile for MI and IHD (both *p*-values for trend < 0.001). Higher odds of IHD and MI were observed for the two main methods of opium consumption (oral and smoking), but oral administration had the highest odds ratio. In addition to adjusting for smoking, we performed a sensitivity analysis in smokers and non-smokers, since some of the increased odds of IHD and MI probably are driven from residual confounding from smoking or its interaction effects with opium use. We found that, use of opium alone (in the non-smokers) increased odds of IHD (adjusted odds ratio (OR_adj_): 1.35, 95%CI 1.01 to 1.80) and MI (adjusted odds ratio (OR_adj_): 1.70, 95%CI 1.09 to 2.66), however, opium consumption accompanied with cigarette smoking (in the smokers) increased the odds of IHD (odds ratio: 1.80, 95%CI 1.28 to 2.53) and MI (odds ratio (OR): 1.97, 95%CI 1.24 to 3.12) beyond using opium alone. However, despite these increases, the interaction between opium consumption and cigarette smoking was not statistically significant (Additional file 2: eTable 1). Also, we investigated the interaction between opium use and gender, which was not statistically significant (Additional file 2: eTable 1). Additionally, to determine the direction and magnitude of the non-differential misreporting bias, we compared the results of simple bias analysis with those of the conventional results assuming that the specificity and sensitivity of the self-reported opium consumption in both healthy and patient groups are 90% (see other assumptions in the Additional file 3: eTable 2 and Table 3).

**Table 3:** Association of opium consumption with myocardial infarction and Ischemic heart diseases (n = 9952*)

	Crude model	Adjusted model 1	Adjusted model 2	Adjusted model 3
	OR (95%Ci)^**a**^	OR(95%Ci)^**b**^	OR (95%Ci)^**c**^	OR(95%Ci)^**d**^
Ischemic heart diseases				
Opium consumption				
yes	1.68 (1.44–1.96)	1.51 (1.24–1.82)	1.57 (1.28–1.93)	1.51 (1.22–1.86)
no	1	1	1	1
Duration of opium consumption				
Non-user	1	1	1	1
≤ 5 year	0.95 (0.66–1.36)	0.94 (0.64–1.36)	0.97 (0.66–1.42)	0.84 (0.57–1.25)
6–10 year	1.15 (0.83–1.60)	1.23 (0.87–1.75)	1.29 (0.90–1.86)	1.28 (0.89–1.85)
11/15 year	1.66 (1.18–2.33)	1.86 (1.28–2.70)	1.99 (1.35–2.94)	2 (1.33–2.99)
16/20 year	1.90 (1.42–2.55)	1.90 (1.37–2.64)	2.07 (1.46–2.92)	2.11 (1.48–3.01)
> 20 year	2.74 (2.18–3.45)	1.82 (1.39–2.37)	1.94 (1.45–2.60)	1.99 (1.47–2.70)
Route of opium consumption				
Non-user	1	1	1	1
Smoking	1.60 (1.37–1.87)	1.45 (1.20–1.76)	1.50 (1.22–1.85)	1.47 (1.18–1.82)
Oral consumption	3.30 (2.12–5.13)	2.33 (1.46–3.74)	2.46 (1.50–4.02)	2.30 (1.36–3.89)
Myocardial infarction				
Opium consumption				
yes	3.35 (2.64–4.25)	2.07 (1.56–2.73)	1.93 (1.42–2.63)	1.79 (1.31–2.45)
no	1	1	1	1
Duration of opium consumption				
Non-user	1	1	1	1
≤ 5 year	1.55 (0.89–2.71)	1.19 (0.67–2.11)	1.14 (0.64–2.04)	0.98 (0.54–1.79)
6–10 year	1.69 (1–2.85)	1.22 (0.70–2.12)	1.19 (0.67–2.08)	1.20 (0.70–2.07)
11-15 year	4.10 (2.64–6.37)	3.07 (1.91–4.93)	2.97 (1.80–4.89)	2.92 (1.76–4.84)
16–20 year	4.67 (3.18–6.86)	3.064 (2.01–4.67)	3.04 (1.94–4.76)	3.11(1.97–4.89)
> 20 year	5.08 (3.65–7.08)	2.29 (1.57–3.33)	2.20 (1.45–3.35)	2.15 (1.41–3.28)
Route of opium consumption				
Non-user	1	1	1	1
Smoking	3.23 (2.53–4.12)	2.02 (1.52–2.68)	1.89 (1.38–2.60)	1.77 (1.30–2.42)
Oral consumption	5.54 (2.99–10.28)	2.81(1.47–5.35)	2.52 (1.28–4.95)	2.12 (1.04–4.30)

^*^Excluding 38 individuals who have started opium after their illness

^a^The baseline model is stratified on the status of opium consumption

^b^The adjusted model 1 is adjusted for confounding variables age (continuous variable), gender (male/ female) and education years (continuous variable)

^C^The adjusted model 2 has additional adjustment for confounding the variables related to lifestyle (cigarette smoking, alcohol drinking and hookah consumption) and physical activity level (continuous variable)

^d^The adjusted model 3 has additional adjustment for hypertension (yes/no), hypercholesterolemia (continuous variable), body mass index (continuous variable) and diabetes mellitus (yes/no), Triglycerides (continuous variable), LDL cholesterol (continuous variable), HDL cholesterol (continuous variable), S.G.O.T (AST) (continuous variable), S.G.P.T (ALT) (continuous variable), Alkaline phosphatase (continuous variable)

The adjusted odds ratios for this bias about IHD and MI are 2.14 and 5.17, respectively. These Odds ratios were reported in the crude logistics model before adjusting for this bias as 1.68 and 3.35, respectively. Based on this analysis (on condition of the accuracy of the values assigned to the bias parameters), percent biases, were − 22% for IHD and − 35% for MI, indicating that the odds ratio increased or, on the other words, there was 25 and 35% error towards null before adjusting for this bias (see Additional file 4: eTable 4 for more details on conducting a simple bias analysis).

## Discussion

The present study is a crass-sectional study aimed at assessment of the association between opium use and cardiovascular diseases in the participants of the Rafsanjan Cohort Study, an area in the southeast of Iran with a relatively high prevalence of opium use. According to our findings, 23.81% of the Rafsanjani adult population (46.19% of men and 4.27% of women) reported opium use at least once per week for 6 months. The main finding of this study was that cardiovascular diseases were considerably more common among opium users (even after adjusting for some potential confounding variables) compared with non-users with a dose-response relationship. Our results on dose–response relationships between the risk of cardiovascular disease and duration of opium consumption strengthened the conclusion that opium use was directly associated with an increased risk of cardiovascular disease. This finding was consistent with those of similar studies. Niaki et al. showed that opium consumption was a significant risk factor of MI with an adjusted odds ratio of 26.3 [9]. Sadeghian et al. presented the opium abuse as a major risk factor of ischemic heart disease [29]. In another study on 556 patients with MI, opium addiction was found in 19% of the patients versus 2.8% of the general population [19]. In a similar study on patients with coronary artery disease (CAD) confirmed with angiography, an association between opium addiction and the development of CAD was found in the male patients [30]. Another angiographic study on 2405 patients, demonstrated the higher prevalence of vascular involvement in addicts in comparison to non-addict people [29]. Also, a review study came to the conclusion that opium consumption may be associated with high risk of the other chronic diseases, such as cardiovascular diseases [31]. In accordance with this finding, Hosseini et al. observed a dose-response relationship between dose of opium and the Gensini score (β = 0.27, *p* = 0.04), after adjustment for potential confounders [32]. As expected, in all routes of opium consumption, there was a direct relation between opium use and cardiovascular disease; meantime, another result of our study was that oral administration of opium was associated with higher odds of cardiovascular disease. This finding was in accordance with the report of Mohammadi et al. showed that oral opium administration accelerated atherosclerosis formation in hypercholesterolemia rabbits [33]. Hosseini et al. observed no significant differences between the routes of opium administration (inhalation vs. oral) regarding the extent and severity of CAD [32]. Since several reports suggested lead poisoning in Iranian opium addicts, it is possible that lead in opium increases the risk of CVDs in opium users. The opium adulterated with lead is a new source of lead poisoning in Iran, where the opium abuse is relatively frequent [6]. Chemical analysis of the opium has confirmed this claim. Generally, consumption of contaminated drugs have been reported as a source of exposure to toxins such as arsenic and lead [34]. Although, the mechanisms of the effects of opium administration on cardiovascular diseases are not precisely known and required to be further investigated, a related research has examined the level of blood lead and mortality risk in the general population of the United States using mortality follow-up data for participant ≥40 years old from NHANES III. The result of this large cohort study indicated the association of increased risk of death from all causes, cardiovascular disease, and cancer with the elevated levels of blood lead. Especially for mortality due to CVDs, there was also a pattern of the increasing risk of the disease with the increasing level of blood lead [35]. Lead-related atherosclerosis could be elucidated using several mechanisms, including increases in blood pressure, impairment of renal function, induction of oxidative stress, inflammation, and endothelial dysfunction [36]. Adequate mechanistic studies have not been performed on the effects of chronic use of opium, so further research is needed to describe the possible mechanisms and check the accuracy of the findings. At conclude, the methods of opium consumption may fewer is considered, while could hence the risk of CVD.

Furthermore, our supplementary analysis showed that opium consumption accompanied by cigarette smoking was associated with higher odds of IHD and MI, compared with using opium alone. However, the interaction between opium consumption and cigarette smoking was not statistically significant. Also, we investigated the interaction between opium use and gender, which was not statistically significant, too.

This study has strengths and limitations. One of the main strengths of our study is its population-based nature with a large sample size, extensive data collection for the exposure of interest (opium) and potential confounders (e.g. cigarette smoking, age, sex and etc.).

However, the study has some limitations too. For instance, it is possible that a number of people have not completely reported their status of opium consumption. On the other hand, there may be also some degrees of misclassification due to self-reporting and recall biases. According to the results of various studies, data of opium use based on self-reporting may cause misreporting when compared with biological tests. So, this type of studies is susceptible to measurement errors such as self-reporting bias, which possibly leads to some deviations from reality (incidence of bias in estimates) [37–41]. However, the amount of this self-reporting bias depends on sex, age, type of substances, geographical area and the under-study population. It can be resulted from the differences in social and cultural beliefs for various individuals and regions [42–44]. Fortunately, we believe that the validity of the data on opium use in our population is relatively high, probably because opium consumed in this population with lower social stigma. In a similar study, Abnet et al. reported a high rate of sensitivity of opium use in a population among Turkmen in the north of Iran [28]. It has been demonstrated that the Turkmen population, use opium as a traditional medicine with low social stigma.

Although, in our study the results of simple bias analysis showed that the direction and magnitude of this bias are probably towards null and the adjusted odds ratio for this bias about IHD and MI is stronger than that of the conventional result, the accuracy of the results of this model is strongly influenced by the accuracy of bias parameters [27]. Since no internal validation study has been performed in this population to assess the self-reporting of opium use, we used the results of an external validation study [28] and guess based on existing conditions [22] to determine the bias parameters that may be not accurate. Therefore, it is suggested to conduct an internal validation study on our population to examine the magnitude and direction of this bias more accurately.

Although, we excluded the patients who reported opium use after their illness, it is possible that some participants have mistakenly reported their age of start of opium consumption and/or the age of their illness diagnosis, as a result of recall bias. On the other words, they have started opium consumption after development of the CVDs to suppress angina symptom and so, opium use is not a risk factor. Accordingly, it is suggested that this relationship be reconsidered in the follow-up phase of this prospective study.

## Conclusions

In conclusion, our results shows that opium consumption not only has no ameliorating effect on CVDs; it may have some adverse effects on these diseases. Therefore, people should be informed about the hazardous effects of opium consumption on cardio-metabolic diseases.

## Supplementary Information

**Additional file 1:** Figure 1.

**Additional file 2:** eTable 1.

**Additional file 3:** eTable 2 and 3.

**Additional file 4:** eTable 4.

## Data Availability

The datasets used during the current study are available on the Persian Adult Cohort Study Center, Rafsanjan University of Medical Sciences, Iran. The data is not available publicly. However, upon a reasonable request, the data can be obtained from the authors.

## Abbreviations

RCS: The Rafsanjan cohort study
PERSIAN: Prospective epidemiological research studies in IrAN
UNODC: United Nations Office of drugs and crime
CVDs: Cardiovascular diseases
WHO: World Health Organization
BMI: Body mass index
CI: Cardiac ischemic
MI: Myocardial infarction
HDL: High density lipoprotein
LDL: Low density lipoprotein
OR: Odds ratio

## References

[1] Malekinejad M, Vazirian M (2012). Transition to injection amongst opioid users in Iran: implications for harm reduction. Int J Drug Policy.

[2] United Nations Office on Drugs, Crime. World drug report 2010. United Nations Publications; 2010.

[3] Merz F (2018). United Nations Office on drugs and crime: world drug report 2017. 2017. SIRIUS-Zeitschrift für Strategische Analysen.

[4] United Nations Office on Drugs and Crime (UNODC), World Drug Report 2017. Vienna. http://www.unodc.org/wdr2017/press/WDR_Press_Release.pdf. Accessed 31 July 2018.

[5] Shahouzehi B, Shokoohi M, Najafipour H (2013). The effect of opium addiction on serum adiponectin and leptin levels in male subjects: a case control study from Kerman coronary artery disease risk factors study (KERCADRS). EXCLI J.

[6] Soltaninejad K, Shadnia S. Lead poisoning in opium abuser in Iran: a systematic review. Int J Prev Med. 2018;9. 10.4103/ijpvm.IJPVM_22_17 PMC5787876 29416839

[7] Nemati A, Jafari S, Afshari M, Dahmardeh S, Tabrizian K (2016). Comparing blood lead level among oral/inhaled opium addicts with a non-addict control group in the southeast of Iran. Addict Health..

[8] Salehi H, SAYADI AAR, Tashakori M, Yazdan DR, Soltanpour N, Sadeghi H (2009). Comparison of serum lead level in oral opium addicts with healthy control group.

[9] Niaki MRK, Hamid M, Farshidi F, Mohammadpour M, Omran MTS (2013). Evaluation of the role of opium addiction in acute myocardial infarction as a risk factor. Caspian J Int Med.

[10] Mayosi BM, Cupido B, Lawrenson J. Cardiovascular Diseases. Hunter's Tropical Medicine and Emerging Infectious Diseases: Elsevier; 2020. p. 8–15.

[11] Read SH, Wild SH (2020). Prevention of premature cardiovascular death worldwide. Lancet.

[12] Nowbar AN, Gitto M, Howard JP, Francis DP, Al-Lamee R (2019). Mortality from ischemic heart disease: analysis of data from the World Health Organization and coronary artery disease risk factors from NCD risk factor collaboration. Circulation.

[13] Safaii N, Kazemi B (2010). Effect of opium use on short-term outcome in patients undergoing coronary artery bypass surgery. Gen Thorac Cardiovasc Surg.

[14] Gozashti MH, Mohammadzadeh E, Divsalar K, Shokoohi M (2014). The effect of opium addiction on thyroid function tests. J Diab Metab Disord.

[15] Najafipour H, Masoomi M, Shahesmaeili A, Haghdoost AA, Afshari M, Nasri HR, et al. Effects of opium consumption on coronary artery disease risk factors and oral health: results of Kerman coronary artery disease risk factors study a population-based survey on 5900 subjects aged 15-75 years. Int J Prev Med. 2015;6. 10.4103/2008-7802.157470 PMC4455126 26097671

[16] Van Den Brink OW, Delbridge LM, Rosenfeldt FL, Penny D, Esmore DS, Quick D (2003). Endogenous cardiac opioids: enkephalins in adaptation and protection of the heart. Heart Lung Circ.

[17] Masoomi M, Azdaki N, Shahouzehi B (2015). Elevated plasma homocysteine concentration in opium-addicted individuals. Addict Health.

[18] Najafi M, Sheikhvatan M (2012). Plausible impact of dietary habits on reduced blood sugar in diabetic opium addicts with coronary artery disease. Int Cardiovasc Res J.

[19] Bafghi SS, Rafiei M, Bahadorzadeh L, Namayeh S, Soltani M, Andishmand MMA (2005). Is opium addiction a risk factor for acute myocardial infarction?. Acta Med Iranica..

[20] Amin-Esmaeili M, Rahimi-Movaghar A, Sharifi V, Hajebi A, Radgoodarzi R, Mojtabai R (2016). Epidemiology of illicit drug use disorders in Iran: prevalence, correlates, comorbidity and service utilization results from the Iranian mental health survey. Addiction..

[21] Poustchi H, Eghtesad S, Kamangar F, Etemadi A, Keshtkar AA, Hekmatdoost A (2018). Prospective epidemiological research studies in Iran (the PERSIAN cohort study): rationale, objectives, and design. Am J Epidemiol.

[22] Hakimi H, Ahmadi J, Vakilian A, Jamalizadeh A, Kamyab Z, Mehran M (2020). The profile of Rafsanjan Cohort Study.

[23] Kapoor L. Opium poppy: botany, chemistry, and pharmacology: CRC Press; 1995.

[24] Shakeri R, Kamangar F, Mohamadnejad M, Tabrizi R, Zamani F, Mohamadkhani A, et al. Opium use, cigarette smoking, and alcohol consumption in relation to pancreatic cancer. Medicine. 2016;95(28). 10.1097/MD.0000000000003922 PMC4956779 27428185

[25] Greenland S, Pearl J, JMJE R (1999). Causal diagrams for epidemiologic research.

[26] Jewell NP. Statistics for epidemiology: CRC Press; 2003.

[27] Lash TL, Fox MP, Fink AK. Applying quantitative bias analysis to epidemiologic data: Springer Science & Business Media; 2011.

[28] Abnet CC, Saadatian-Elahi M, Pourshams A, Boffetta P, Feizzadeh A, Brennan P (2004). Reliability and validity of opiate use self-report in a population at high risk for esophageal cancer in Golestan, Iran. Cancer Epidemiol Prev Biomark.

[29] Sadeghian S, Darvish S, Davoodi G, Salarifar M, Mahmoodian M, Fallah N (2007). The association of opium with coronary artery disease. Eur J Cardiovasc Prev Rehabil.

[30] Sadeghian S, Graili P, Salarifar M, Karimi AA, Darvish S, Abbasi SH (2010). Opium consumption in men and diabetes mellitus in women are the most important risk factors of premature coronary artery disease in Iran. Int J Cardiol.

[31] Masoudkabir F, Sarrafzadegan N, Eisenberg MJ (2013). Effects of opium consumption on cardiometabolic diseases. Nat Rev Cardiol.

[32] Hosseini SK, Masoudkabir F, Vasheghani-Farahani A, Alipour-Parsa S, Fathollahi MS, Rahimi-Foroushani A (2011). Opium consumption and coronary atherosclerosis in diabetic patients: a propensity score-matched study. Planta Med.

[33] Mohammadi A, Darabi M, Nasry M, Saabet-Jahromi M-J, Malek-Pour-Afshar R, Sheibani H (2009). Effect of opium addiction on lipid profile and atherosclerosis formation in hypercholesterolemic rabbits. Exp Toxicol Pathol.

[34] Jalili M, Azizkhani R (2009). Lead toxicity resulting from chronic ingestion of opium. Western J Emerg Med.

[35] Schober SE, Mirel LB, Graubard BI, Brody DJ, Flegal KM (2006). Blood lead levels and death from all causes, cardiovascular disease, and cancer: results from the NHANES III mortality study. Environ Health Perspect.

[36] Navas-Acien A, Guallar E, Silbergeld EK, Rothenberg SJ (2007). Lead exposure and cardiovascular disease—a systematic review. Environ Health Perspect.

[37] Gryczynski J, Schwartz RP, Mitchell SG, O’Grady KE, Ondersma SJ (2014). Hair drug testing results and self-reported drug use among primary care patients with moderate-risk illicit drug use. Drug Alcohol Depend.

[38] Harrison L, Hughes A (1997). The validity of self-reported drug use: improving the accuracy of survey estimates. NIDA Res Monogr.

[39] Yacoubian GS, VanderWall KL, Johnson RJ, Urbach BJ, Peters RJ (2003). Comparing the validity of self-reported recent drug use between adult and juvenile arrestees. J Psychoactive Drugs.

[40] Tourangeau R, Yan T (2007). Sensitive questions in surveys. Psychol Bull.

[41] Magura S, Kang S-Y (1996). Validity of self-reported drug use in high risk populations: a meta-analytical review. Substance Use Misuse.

[42] Rashidian H, Hadji M, Marzban M, Gholipour M, Rahimi-Movaghar A, Kamangar F, Malekzadeh R, Weiderpass E, Rezaianzadeh A, Moradi A, Babhadi-Ashar N (2017). Sensitivity of self-reported opioid use in case-control studies: Healthy individuals versus hospitalized patients. PloS One..

[43] Yacoubian GS, VanderWall KL, Johnson RJ, Urbach BJ, Peters RJ (2003). Comparing the validity of self-reported recent drug use between adult and juvenile arrestees. J Psychoact Drugs..

[44] Fendrich M, Mackesy-Amiti ME, Johnson TP (2008). Validity of self-reported substance use in men who have sex with men: comparisons with a general population sample. Ann Epidemiol..

