# Supplementary figures and images for: Effect of opium consumption on cardiovascular diseases – a cross- sectional study based on data of Rafsanjan cohort study
Source: BMC Cardiovasc Disord. 2021 Jan 2;21:2. doi: 10.1186/s12872-020-01788-4 (PMC7778811; doi:10.1186/s12872-020-01788-4)

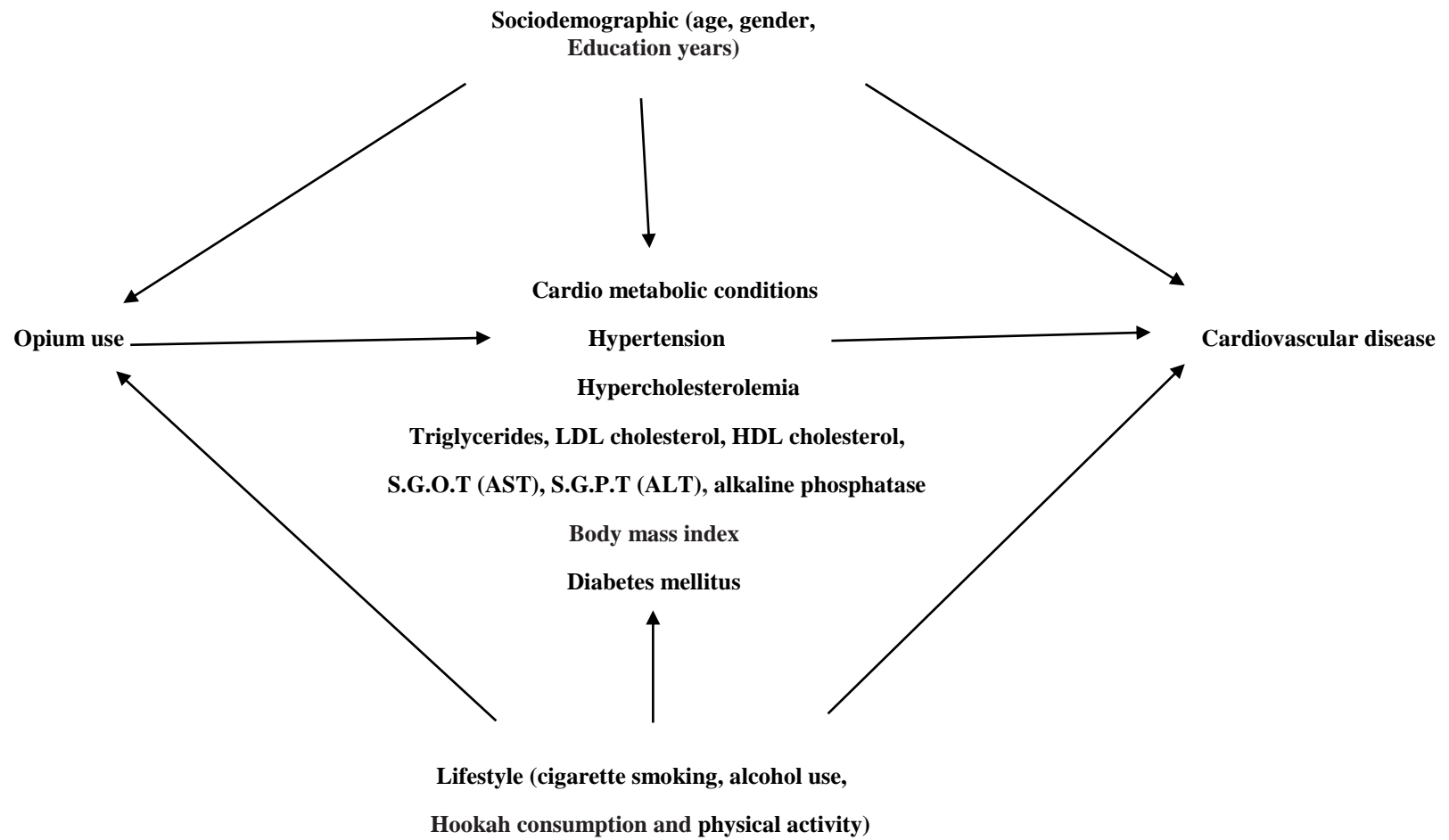

Figure 1. Causal diagram.

Supplement: Supplementary file 1 — Additional file 1: Figure 1. [file 12872_2020_1788_MOESM1_ESM.pdf]
