# Supplementary material for: Effect of opium consumption on cardiovascular diseases – a cross- sectional study based on data of Rafsanjan cohort study
Source: BMC Cardiovasc Disord. 2021 Jan 2;21:2. doi: 10.1186/s12872-020-01788-4 (PMC7778811; doi:10.1186/s12872-020-01788-4)
Supplement: Supplementary file 2 — Additional file 2: eTable 1. [file 12872_2020_1788_MOESM2_ESM.docx]

| eTable 1: Association of opium consumption with myocardial infarction and Ischemic heart diseases according to sex and smoking (n=9,952^a^). | | |
| --- | --- | --- |
|  | **Myocardial infarction** | **Ischemic heart diseases** |
|  | **OR_adj_^b^ (95%CI)** | **OR_adj_ ^b^(95%CI)** |
| **Smoker(n= 2,524)**^c^ | |  |
| **Opium consumption** | |  |
| yes | 1.97 ( 1.24- 3.12) | 1.80(1.28 -2.53) |
| no | 1 | 1 |
| **Non-smoker(n=7,427)** | |  |
| **Opium consumption** | |  |
| yes | 1.70(1.09- 2.66) | 1.35(1.01- 1.80) |
| no | 1 | 1 |
| **interaction between smoking and opium use** | 1.35(.87 - 2.10) | 1.28(.84 -1.95) |
| **Male** | |  |
| **Opium consumption** |  |  |
| yes | 1.70(1.20 -2.41) | 1.64(1.27- 2.12) |
| no | 1 | 1 |
| **Female** |  |  |
| **Opium consumption** |  |  |
| yes | 2.13(1.04- 4.36) | 1.13(.72-1.77) |
| no |  |  |
| **interaction between gender and opium use** | 0.66(0.40 -1.09) | 1.052(0.49 - 2.26) |
| ^a^ Excluding 38 who have started opium after their illness  ^b^ The adjusted model 3  ^c^ Those who had started smoking before illness | | |
