# Supplementary material for: Effect of opium consumption on cardiovascular diseases – a cross- sectional study based on data of Rafsanjan cohort study
Source: BMC Cardiovasc Disord. 2021 Jan 2;21:2. doi: 10.1186/s12872-020-01788-4 (PMC7778811; doi:10.1186/s12872-020-01788-4)
Supplement: Supplementary file 3 — Additional file 3: eTable 2 and 3. [file 12872_2020_1788_MOESM3_ESM.docx]

| eTable 3. Simple bias analysis of the opium use- Ischemic heart diseases odds ratios under various assumptions about the sensitivity (Se) and specificity (Sp) of self-reported opium use among healthy and patient groups. | | | | | | |
| --- | --- | --- | --- | --- | --- | --- |
| Healthy group | | | | | Patient group | |
| 0.8 | 0.9 | 0.8 | 0.9 | Se | Sp | Se |
| 0.8 | 0.8 | 0.9 | 0.9 | Sp |  |  |
| 8.38 | 9.85 | 1.82 | **2.14** |  | 0.9 | 0.9 |
| 10.17 | 11.95 | **2.21** | 2.6 |  | 0.9 | 0.8 |
| 4.76 | **5.59** | 1.03 | 1.22 |  | 0.8 | 0.9 |
| **5.78** | 6.78 | 1.26 | 1.47 |  | 0.8 | 0.8 |
| Under non-differential misclassification (bolded cells), bias corrected odds ratios are always further away from the null. | | | | | | |

| eTable 2. Simple bias analysis of the opium use- Myocardial infarction odds ratios under various assumptions about the sensitivity (Se) and specificity (Sp) of self-reported opium use among healthy and patient groups. | | | | | | |
| --- | --- | --- | --- | --- | --- | --- |
| Healthy group | | | | | Patient group | |
| 0.8 | 0.9 | 0.8 | 0.9 | Se | Sp | Se |
| 0.8 | 0.8 | 0.9 | 0.9 | Sp |  |  |
| 19.68 | 23.12 | 4.40 | **5.17** |  | 0.9 | 0.9 |
| 26.20 | 30.78 | **5.85** | 6.88 |  | 0.9 | 0.8 |
| 14.73 | **17.31** | 3.29 | 3.87 |  | 0.8 | 0.9 |
| **19.62** | 23.05 | 4.38 | 5.15 |  | 0.8 | 0.8 |
| Under non-differential misclassification (bolded cells) bias corrected Odds ratios are always further away from the null. | | | | | | |
