# Supplementary material for: Effect of opium consumption on cardiovascular diseases – a cross- sectional study based on data of Rafsanjan cohort study
Source: BMC Cardiovasc Disord. 2021 Jan 2;21:2. doi: 10.1186/s12872-020-01788-4 (PMC7778811; doi:10.1186/s12872-020-01788-4)
Supplement: Supplementary file 4 — Additional file 4: eTable 4. [file 12872_2020_1788_MOESM4_ESM.docx]

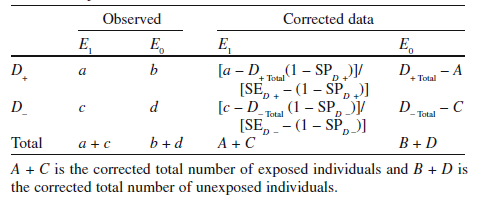


**Figure 2.** Equation (taken from applying quantitative bias analysis to epidemiologic data book) used for calculating expected true data given the observed data with exposure misclassification: *Corrections Using Sensitivity and Specificity*

| eTable 4: Correction for misclassification of opium use in the study of the effects of opium consumption on myocardial infarction and ischemic heart diseases assuming non-differential misclassification | | | | | | | | |
| --- | --- | --- | --- | --- | --- | --- | --- | --- |
| **Ischemic heart diseases** | | | | **Myocardial infarction** | | | |  |
| Corrected data | | Observed | | Corrected data | | Observed | |  |
| **Non-user** | **user** | **Non-user** | **user** | **Non-user** | **user** | **Non-user** | **user** |  |
|  |  |  |  |  |  |  |  | Se = 90%, Sp = 90% |
| 592.125 | 240.875 | 557 | 276 | 142.125 | 140.875 | 142 | 141 | case |
| 7614.75 | 1447.25 | 6998 | 2064 | 8064.75 | 1547.25 | 7413 | 2199 | control |
|  | 2.14 |  | 1.68 |  | 5.17 |  | 3.35 | OR |
| Sensitivity (Se) = probability someone exposed is classified as exposed  Specificity (Sp) = probability someone unexposed is classified as unexposed | | | | | | | | |
